# The matching effect in persuasive communication about lockdown

**DOI:** 10.3389/fpsyg.2022.987114

**Published:** 2022-10-12

**Authors:** Isabella Giammusso, Antonio Aquino, Francesca Romana Alparone, Alberto Mirisola

**Affiliations:** ^1^Department of Psychology, Educational Science and Human Movement, University of Palermo, Palermo, Italy; ^2^Department of Neuroscience, Imaging and Clinical Sciences, University of Chieti-Pescara, Chieti, Italy

**Keywords:** attitude, need for affect, need for cognition, lockdown, Twitter, matching effect

## Abstract

Scientific literature about persuasion has shown that the effectiveness of persuasive communication may depend on the match between the affective or cognitive contents of the message and the affective [(Need for Affect (NFA)] or cognitive [Need for Cognition (NFC)] orientation of the recipient. The present work aims to contribute to studying this effect by considering the context of health-related communication during the SARS-CoV-2 infection. Specifically, we aim to demonstrate that, when the message is characterized by affective and cognitive contents having the same (congruent message) or different valence (incongruent message), the attitude toward the target (i.e., a new lockdown) will be guided by the valence of the contents matching the individual affective/cognitive orientation. A total of 1,003 participants took part in a 2 (Cognitive content message: Positive vs. Negative) × 2 (Affective content message: Positive vs. Negative) factorial design and answered an online questionnaire. Results show that people with high levels of NFA and low levels of NFC report attitudes toward lockdown consistent with the valence of the affective contents. Conversely, attitudes of people with high levels of NFC and low levels of NFA were not influenced by contents that matched their orientation (i.e., cognitive).

## Introduction

In December 2019, a new coronavirus (SARS-CoV-2) emerged in Wuhan, China, sparking an epidemic of an acute respiratory syndrome (COVID-19) in humans (Wang et al., 2020). Within 3 months, the virus had spread to more than 118,000 cases and caused 4,291 deaths in 114 countries, leading the World Health Organization (WHO) to declare a global pandemic. The pandemic has led to a massive global public health campaign to slow the spread of the virus by increasing hand washing, reducing face touching, wearing masks in public, and promoting physical distancing. In Italy, on March 9, 2020, Prime Minister Giuseppe Conte imposed a national lockdown in response to the growing pandemic of COVID-19 in the country. According to the Decree of the Italian President of the Council of Ministers (2020), lockdown implied the cancellation and prohibition of all public events (including funerals, masses, and sports), the closure of non-essential services (e.g., restaurants, pubs), and the online provision of schools and universities services (both teaching and back office). People’s mobility was allowed only for a strict list of necessities (e.g., professions impossible to be performed online, health emergencies, grocery shopping) and upon presentation of certifying documents, while persons infected with the virus were confined at home or treated in dedicated hospital ward, depending on their conditions. However, despite the effectiveness of lockdown in saving human lives (Ferguson et al., 2020; Prem et al., 2020; Cerqueti et al., 2022), this non-pharmaceutical measure had great costs for citizens in terms of isolation and economic losses (The Economist, 2020). Therefore, to guarantee a successful implementation of the lockdown, it was fundamental to obtain people’s compliance by effectively communicating with them. In such a scenario, persuasive health communication plays a prominent role. Social psychologists define persuasion as a communication finalized to produce changes in attitudes and the related behaviors (e.g., reducing smoking, purchases, voting; Eagly and Chaiken, 1993; Cacioppo et al., 1994). In the context of the implementation of preventive measures against COVID-19, evidence showed how a positive attitude toward them favors people’s compliance (Ammar et al., 2020; Kim et al., 2020; Zhang et al., 2020; Wollast et al., 2021).

Persuasive communication has been so important in inducing positive attitude in citizen toward strategies to reduce COVID-19 spreading that even WHO edited and published guidelines for governments (i.e., World Health Organization [WHO], 2020), but also for specific social actors, such as journalists (World Health Organization Regional Office for Europe, 2021). These guidelines emphasize the importance of proactive communication that should be engaging for the public and clear enough so non-experts can understand it. WHO has acknowledged the importance of communication on social media about preventive measures, given that social platforms have assumed the role of an online public arena where people obtain information about COVID-19 and may prompt positive (or negative) attitudes toward these measures (Pennycook et al., 2020). Therefore, it becomes essential to identify the features of social communication that might influence attitudes toward preventive strategies in a health context as that against COVID spread.

In this context, it is noteworthy that the affective-cognitive content of the message is a key feature of persuasive communication. In attitude literature, affective appeals refer to communication emphasizing the positive or negative feelings and emotions that an individual associate with an attitude object (e.g., going to the gym makes me happy). Instead, cognitive appeals refer to communication emphasizing beliefs about positive or negative attributes of an attitude object (e.g., going to the gym prevents cardiovascular disease; Ostrom, 1969; Breckler, 1984; Crites et al., 1994). Related to the affective-cognitive content issue is the research on the structural matching effect, considered a cornerstone in the literature on persuasion. Indeed, it has been observed in several studies (e.g., Fabrigar and Petty, 1999; Huskinson and Haddock, 2004; Haddock et al., 2008; Di Plinio et al., 2022) that people’s preference for affective information predicted greater persuasion in response to an affect-based (but not cognition-based) message, whereas the preference for cognitive information predicted greater persuasion in response to a cognition-based (but not affect-based) message.

The matching effect is usually referred to the structure of attitudes, which may be predominantly based on feelings and/or beliefs (e.g., Katz and Stotland, 1959; Hovland and Rosenberg, 1960; Zajonc and Markus, 1982; Cacioppo et al., 1989) that an attitude object evokes in the evaluators (e.g., Ostrom, 1969; Crites et al., 1994). Research has demonstrated that people differ in the degree to which their evaluations are guided by affective and cognitive message contents (see Haddock and Maio, 2019, for a review). These orientations are identified as individual differences in need for affect (NFA; Maio and Esses, 2001) and need for cognition (NFC; Cacioppo and Petty, 1982). NFA describes the degree to which people prefers to approach or avoid situations that are emotion-inducing (Maio and Esses, 2001). People high in NFA are motivated to understand their own and others’ emotions and tend to use emotional information in attitude formation and attitude change (Huskinson and Haddock, 2004). NFC describes individual differences in the tendency to engage in and enjoy complex activities requiring cognitive effort. People high in NFC are more likely to seek information about an object’s attributes before evaluating it (Haugtvedt et al., 1992). Research has demonstrated that NFA and NFC predict outcomes related to attitude formation and attitude change, as in the aforementioned structural matching effect.

However, matching effect studies have so far focused on the effect of messages comprising either cognitive or affective contents, without considering the impact of a message comprising mixed contents. Therefore, the present research aims to study the matching effect in the context of more complex communication, in which the message is characterized by both affective and cognitive contents, as is often the case in informal communication, and in social networks. Furthermore, affective and cognitive contents may be congruent in terms of valence, so the message frame will be either positive or negative, but they may also be incongruent in terms of valence (e.g., positive affective and negative cognitive contents and vice versa). In the latter case, the target audience will be faced with a mixed message. The question then arises as to whether the persuasive effect of such a message will depend solely on the correspondence between people’s affective/cognitive orientation and the affective/cognitive content of the message (i.e., the classic matching effect) or whether such persuasive effect also takes into account the positive/negative valence of arguments not matching the individuals’ orientation. We are unaware of studies examining the matching effect in such a situation. Thus we aimed to fill this gap in the literature. Our goal was to test if people’s attitudes would still be influenced by the matching between the message and the individual orientation in the case of messages combining affective and cognitive contents of different valences:

**Research question:** Will the content matching the individual orientation influence people’s attitude when the message is characterized by mixed contents (affective and cognitive) having the same (e.g., positive affective and cognitive contents) or different (e.g., positive affective and negative cognitive) valence? This research question will be particularly relevant when the message content is incongruent (e.g., positive affective and negative cognitive contents and vice versa).

According to this research question, we hypothesized that:

**H1a**: Regardless of the valence of cognitive contents, participants with high NFA and low NFC exposed to persuasive lockdown messages characterized by positive affective content would have reported more positive attitudes toward lockdown. The same participants would have reported more negative attitudes toward lockdown when exposed to persuasive lockdown messages characterized by negative affective content.

**H1b**: Regardless of the valence of affective contents, participants with high NFC and low NFA exposed to persuasive lockdown messages characterized by positive cognitive content would have reported more positive attitudes toward lockdown. The same participants would have reported more negative attitudes when exposed to persuasive lockdown messages characterized by negative cognitive content.

## Materials and methods

### Participants

Considering that the effect size of the moderation effect of NFA and NFC on the relation between persuasive ambivalent messages on attitudes is unknown, we planned the sample size to achieve a power of 0.80, an α of 0.05, and assuming a medium effect size (*f*^2^ = 0.01) (Aguinis et al., 2005). The estimated sample size was 981. Participants were collected online through snowball sampling, and we reached 1,220 answers. We eliminated 217 participants that failed the instructional manipulation check. This percentage was lower than that found in large online studies during the pandemic (e.g., Bago et al., 2022). Therefore, the final sample was composed of 1,003 Italian participants (36.25% males, 63.75% females, *M*_*age*_ = 28.22, SD_*age*_ = 11.96).

### Procedure and measures

We tested our hypotheses by using a 2 (affective positive vs. affective negative) × 2 (cognitive positive vs. cognitive negative) factorial design. Participants were randomized to the experimental conditions based on the season they were born in:

- cognitive and affective positive condition: 345 participants;
- cognitive and affective negative condition: 314 participants;
- cognitive positive and affective negative condition: 336 participants;
- cognitive negative and affective positive condition: 225 participants.

They were presented with an informed consent form, and then they completed the first part of the online questionnaire that included NFA and NFC measures. Then, after reading one of the four persuasive Twitter pages, participants answered the second part of the questionnaire assessing their attitudes toward a new lockdown. Finally, they filled out a socio-demographic form.

For what concerns the experimental conditions, each participant was presented with 1 of 4 possible fabricated Twitter pages (Post’s and comments’ contents in their original version with translations in English are available in the Supplementary material). Each of them was characterized by the same structure: a tweet affirming that in the case of spread of a new COVID-19 variant resistant to the vaccine, a new total lockdown will be inevitable, accompanied by six comments. The main tweet was presented as written by a fake profile named “COVID-19 Prevention Network,” while the comments were randomly attributed to the fake profiles of three women and three men. The four conditions were created by presenting comments resulting from the combination of the valence of affective and cognitive contents:

• cognitive and affective positive condition: three comments containing positive cognitive wording (e.g., *I think a new lockdown would be really useful because data suggests it has been effective in limiting the contagion problem*) and three comments containing positive affective wording (e.g., *I would be happy to go back to lockdown because during the first I felt more relaxed than usual*);
• cognitive and affective negative condition: three comments containing negative cognitive wording (e.g., *I think a new lockdown would be useless because it creates more problems than it solves*) and three comments containing negative affective wording (e.g., *The perspective of a new lockdown saddens me, I will feel so alone*);
• cognitive positive and affective negative condition: three comments containing positive cognitive wording and three comments containing negative affective wording;
• cognitive negative and affective positive condition: three comments containing negative cognitive wording and three comments containing positive affective wording.

All the comments were pre-tested. A set of statements was presented to 31 participants (71.43% female, 28.57% males, *M*_*age*_ = 29.82, SD_*age*_ = 9.91). Participants were asked to rate to what extent each of the proposed comments was positive or negative, affective or cognitive, plausible or implausible on a semantic differential with 7 points scale (from −3 to +3). For the final version of the manipulation materials, we chose the comments with a plausibility score not lower than the theoretical median. Then, for each remaining characteristic (positive/negative, affect/cognition), we chose the statements with the difference from the theoretical median point characterized by the biggest effect size. Following these criteria, we selected the three best comments for each condition such that the cognitive positive condition had the same valence as the affective positive condition [*M*_*d*_ = 0.26, *t*(30) = 1.16, *p* = 0.256] but higher levels in affect/cognition rating [*M*_*d*_ = 3.61, *t*(30) = 9.61, *p* < 0.001]. Likewise, affective positive condition differed from affective negative condition in valence [*M*_*d*_ = 3.43, *t*(29) = 8.17, *p* < 0.001] but not in affect/cognition rating [*M*_*d*_ = 0.07, *t*(29) = 0.30, *p* = 0.763]. Affective negative condition, compared with cognitive negative condition, was equal in valence [*M*_*d*_ = 0.28, *t*(29) = 1.91, *p* = 0.066] but lower in affect/cognition rating [*M*_*d*_ = −3.56, *t*(29) = −9.82, *p* < 0.001]. Finally, cognitive negative condition, compared with cognitive positive condition, was equal in affect/cognition rating [*M*_*d*_ = 0.11, *t*(30) = 0.67, *p* = 0.509] but higher levels of negative valence [*M*_*d*_ = −3.44, *t*(30) = −9.00, *p* < 0.001].

#### Need for affect

Individual preferences in experiencing emotions (NFA) were assessed with the short version of the NFA Questionnaire (NAQ–S; Maio and Esses, 2001) realized by Appel et al. (2012). The English scale comprises 10 Likert items (from 1 *totally disagree* to 7 *totally agree*). We resorted to Leone and Presaghi’s (2007) Italian validation to select the corresponding items. Participants were presented with five items assessing the preference in approaching emotions and the other five assessing the avoidance of emotions on a points Likert scale. Considering that the two factors are not correlated (*r* = 0.01, 95% *CI*: −0.05, 0.07, *p* = 0.73), following Maio and Esses (2001) recommendation, we computed separately the mean scores for emotions approach (NFA-App) and avoidance (NFA-Avo). Consequently, analyses were performed including both NFA-App (Cronbach’s α = 0.73) and NFA-Avo (Cronbach’s α = 0.76) scales.

#### Need for cognition

Individual preferences in experiencing cognitive efforts (NFC) were assessed with the Italian version of the Need for Cognition Scale (NCS; Cacioppo and Petty, 1982) realized by Aquino et al. (2018). The scale comprises 16 Likert items (from 1 *totally disagree* to 7 *totally agree*). Considering that the two factors are correlated (*r* = 0.41, 95% *CI*: 0.36, 0.46, *p* < 0.001), we computed a single mean score for NFC after reversing the score of the item assessing the avoidance dimension (Cronbach’s α = 0.80).

#### Attitude toward lockdown

Overall attitude toward lockdown was assessed through seven points semantic differential with four couples of opposite adjectives (desirable/undesirable; unpleasant/pleasant; bad/good; positive/negative; see Bizer et al., 2006). The order of presentation of the adjectives was randomized. The scale showed good reliability (Cronbach’s α = 0.84). An overall mean score was computed after reversing the score of two couples of adjectives (desirable/undesirable; positive/negative) so that higher scores represented a positive attitude toward lockdown.

## Results

All the statistical analyses were performed with the software R (Version 4.0.3; R Core Team, 2020), using *pequod* (Version 0.0-5, Mirisola and Seta, 2016), *psych* (Version 2.0.12; Revelle, 2020), and *pwr* (Version 1.3-0; Champely, 2020) packages.

### Descriptive statistics and correlations

Means, standard deviations, and Pearson’s correlations between variables are shown in Table 1. Furthermore, the correlation matrix showed that attitude toward lockdown was not correlated with NFC, NFA-App, and NFA-Avo. A one-sample *t*-test comparing the attitude toward lockdown mean score (*M* = 2.38, SD = 1.36) against the theoretical middle point of the scale (3) showed that participants generally reported a negative attitude toward a new lockdown [*t*(1,002) = −37.82, *p* < 0.001, *d* = 0.46]. In line with previous literature, the two dimensions of NFA (Approach and Avoidance) showed only a weak correlation with NFC (*r*_*NFA–App*_ = 0.24, *p* < 0.001; *r*_*NFA–Avo*_ = 0.18, *p* < 0.001) confirming the partial independence of affective and cognitive orientations (see Haddock and Maio, 2019, for a review). Furthermore, NFC, NFA-App, and NFA-Avo were not correlated to the overall attitude toward lockdown, supporting the idea that this relationship could be qualified by the matching effect.

**TABLE 1:** Descriptive statistics and correlations between variables.

	1	2	3	4	5	6	*M*	*SD*
1. NFC	−						4.66	0.82
2. NFA-App	0.24***	−					5.66	0.94
3. NFA-Avo	0.18***	0.01	−				4.46	1.32
4. Attitude toward lockdown	–0.06	0.00	0.00	−			2.38	1.36
5. Age	−0.18***	−0.19***	0.03	–0.03	−		28.22	11.96
6. Education	0.12***	−0.02*	0.10*	0.00	0.20***	−	3.47	0.89

*p < 0.05, ***p < 0.001. NFC, Need For Cognition; NFA-App, Need for Affect—Approach; NFA-Avo, Need for Affect—Avoidance.

### The effect of need for affect, need for cognition, and messages content on attitudes toward lockdown

To examine whether NFA and NFC levels moderated the effect of contents of persuasive messages on attitudes, we used a moderated multiple regression. The dependent variable was the attitude toward lockdown, while NFC, both components of NFA, and the valence of affective and cognitive contents of the message (effect coded with positive content = +1 and negative content = −1) were included as predictors. Sex (effect coded with male = +1 and female = −1), age, and educational level were entered as covariates. We entered all the predictors at Step 1, all the two-way interactions at Step 2, and the three-way interactions were entered at Step 3. The results are reported in Table 2.

**TABLE 2:** Moderated regression analysis.

	Step I		Step II		Step III	
			
Predictors	*B*	*SE*	*B*	*SE*	*B*	*SE*
(Intercept)	3.05***	0.32	2.98***	0.33	2.98***	0.33
Valence of affective contents	–0.02	0.04	0.52*	0.26	0.70*	0.27
Valence of cognitive contents	0.26***	0.04	0.39	0.27	0.44	0.27
NFA-App	0.05	0.05	0.28	0.23	0.38	0.24
NFC	−0.12*	0.06	–0.10	0.06	–0.10	0.06
NFA-Avo	0.01	0.03	–0.23	0.17	−0.38*	0.18
Sex	0.16***	0.05	0.17***	0.05	0.17***	0.05
Age	–0.01	0.00	–0.01	0.00	–0.01	0.00
Educational level	0.02	0.05	0.02	0.05	0.01	0.05
Valence of affective contents × valence of cognitive contents			–0.01	0.04	–0.11	0.27
Valence of affective contents × NFA-App			0.01	0.05	–0.08	0.23
Valence of cognitive contents × NFA-App			0.00	0.05	–0.13	0.24
Valence of affective contents × NFC			−0.12*	0.06	−0.15**	0.06
NFA-App × NFC			–0.05	0.05	–0.07	0.05
Valence of cognitive contents × NFC			–0.03	0.06	–0.04	0.06
Valence of affective contents × NFA-Avo			0.03	0.03	0.55**	0.17
Valence of cognitive contents × NFA-Avo			–0.04	0.03	0.23	0.18
NFC × NFA-Avo			0.05	0.04	0.08*	0.04
Valence of affective contents × valence of cognitive contents × NFA-App					–0.04	0.05
Valence of affective contents × NFA-App × NFC					0.02	0.05
Valence of cognitive contents × NFA-App × NFC					0.03	0.05
Valence of affective contents × valence of cognitive contents × NFA-Avo					0.03	0.03
Valence of affective contents × NFC × NFA-Avo					−0.11**	0.04
Valence of cognitive contents × NFC × NFA-Avo					–0.06	0.04
Valence of affective contents × valence of cognitive contents × NFC					0.02	0.06

NFC, Need For Cognition; NFA-App, Need for Affect—Approach; NFA-Avo, Need for Affect—Avoidance. *p < 0.05, **p < 0.01, ***p < 0.001.

At the Step 1, attitude toward lockdown was predicted by cognitive content, sex, and NFC. A positive cognitive content [*B* = 0.26, *t*(937) = 5.98, *p* < 0.001] and male participants [*B* = 0.16, *t*(937) = 3.53, *p* < 0.001] predicted more positive attitude toward lockdown. Instead, the NFC effect was qualified by the three way interaction Affective contents X NFC X NFA-Avo participants [*B* = −0.11, *t*(937) = 3.53, *p* < 0.001], which at the Step 3 increased significantly model *R*^2^ [*R*^2^ = 0.08; *F*_(7, 920)_ = 2.18, *p* < 0.05]. Notably, neither the assumed three-way interaction Cognitive contents X NFC X NFA-Avo nor NFA-App predicted the new lockdown attitude.

**FIGURE 1:**
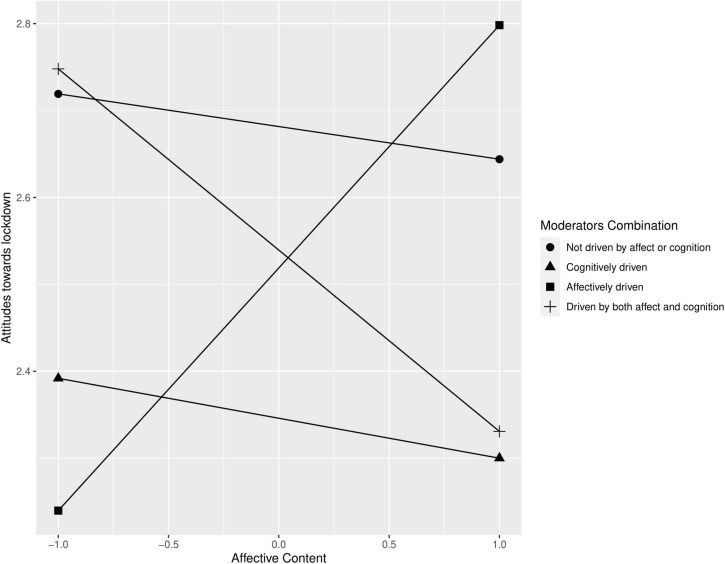
Moderating effect of need for affect (NFA), need for cognition (NFC), and valence of affective contents on attitude toward lockdown.

Simple slope analysis was performed to study the three-way interaction. In particular, we analyzed four simple slopes obtained combining high (+1 SD) and low (−1 SD) levels of NFC and NFA-Avo: not driven by affect or cognition (low NFA-Avo, low NFC), cognitively driven (low NFA-Avo, high NFC), affectively driven (high NFA-Avo, low NFC), and driven by both affect and cognition (high NFA-Avo, high NFC) (see Figure 1). In line with Hp1a, affectively driven participants followed affective messages: more positive affective messages, more affectively driven participants reported positive attitudes toward lockdown [*b* = 0.28, *t*(920) = 2.86, *p* < 0.01]. Differently, participants driven by both affect and cognition reported more negative attitudes toward lockdown [*b* = −0.21, *t*(920) = −2.48, *p* < 0.05] when positive affective content are presented. No significant relationships between affective messages and attitude were found for participants with other level combinations of NFA-Avo and NFC [not driven by affect or cognition: *b* = −0.04, *t*(920) = −0.51, *p* = 0.61; cognitively driven: *b* = −0.05, *t*(920) = −0.51, *p* = 0.61]. Slope difference test (Dawson and Richter, 2006) showed that attitude toward lockdown was more positive for affectively driven participants than for participants driven by both affect and cognition, *t*(920) = −3.67, *p* < 0.001, or participants who are cognitively driven, *t*(920) = 2.33, *p* < 0.05. Other differences between slopes were not significant (see Table 2).

In summarizing, NFA-Avo and NFC levels moderated the relation between the affective content of the message and attitudes toward lockdown. Supporting the hypothesis H1a, people with high levels of NFA-Avo and low levels of NFC showed a matching effect: i.e., more positive attitudes toward lockdown when exposed to positive affective content and more negative attitudes toward lockdown when exposed to negative affective content of the persuasive message, regardless of the valence of cognitive contents (Figure 1). In other words, as expected, people with an affective orientation were influenced by the affective contents of a message, regardless of the cognitive contents’ valence. Differently, we did not find a matching effect of cognitive contents for participants characterized by high levels of NFC and low levels of NFA. Therefore, our hypothesis H1b was not supported. Supplementary analysis showed that attitude toward lockdown was not predicted by both four-way interactions NFA-Avo × NFC X Affective contents × Cognitive contents and NFA-App × NFC × Affective contents × Cognitive contents.

## Discussion

The present research aimed to study the matching effect in the context of messages characterized by mixed contents (e.g., affective and cognitive, positive and negative) considering a specific issue: health-related messages about lockdown spread through Twitter. The structural matching effect (Fabrigar and Petty, 1999; Haddock et al., 2008; Mayer and Tormala, 2010), in fact, has been tested in experiments in which participants were exposed to persuasive messages characterized by either affective or cognitive contents characterized by either congruent or incongruent valence.

In line with the matching effect, we expected that people with an affective orientation would be influenced by the affective contents of a message, regardless of the cognitive contents’ valence. Conversely, we expected that the cognitive contents of a message would have been more influential on people with a cognitive orientation, regardless of the affective contents’ valence. Results partly confirmed our hypothesis.

As expected, we observed that people with high affective (and low cognitive) orientation showed positive attitudes toward lockdown when exposed to messages containing positive affective contents and negative attitudes when exposed to messages containing negative affective contents, and this effect was independent of the congruence of the cognitive content’s valence, as shown by the supplementary analyses. Differently, people with a high cognitive (and low affective) orientation toward lockdown reported attitudes not influenced by cognitive contents of the message. Moreover, the attitude of people with both high affective and high cognitive orientation was more negative in presence of positive affective contents, while the attitude of people not driven by affect or cognition (i.e., low NFA and low NFC) was not influenced by either affective or cognitive contents. This finding is both a direct result of the structural matching effect (Fabrigar and Petty, 1999; Haddock et al., 2008; Mayer and Tormala, 2010) and noteworthy. While the efficacy of the matching of a message with one or more recipient’s characteristics is well-known (see Haddock and Maio, 2019; Teeny et al., 2021), previous studies relied only on simple messages (e.g., positive affective vs. positive cognitive messages; Huskinson and Haddock, 2004). Everyday communication, instead, is characterized by more complex messages. From television news to posts and related comments on social media, messages’ contents (affective or cognitive) and their valence (positive or negative) are often mixed. Therefore, similar to everyday communication, one strength of the present research is the design we used, which included mixed affective and cognitive information. Furthermore, our study showed that, despite the contents’ valence, people’s attitudes are influenced by the content matching their orientation, providing further insights into the structural matching effect.

It is worth noting that our results were obtained only for the avoidance dimension of NFA, by supporting Maio and Esses (2001) suggestions that approach and avoidance are at least somewhat distinct (e.g., Hull, 1952; Miller, 1959; Higgins, 1997). According to the authors, in fact, when approach and avoidance dimensions of NFA showed low correlations, as in the case of the present research, the two dimensions can be considered separately as they are characterized by different correlates. The approach dimension could be particularly relevant in a context where people are expected to feel positive emotions, whereas avoidance could be particularly relevant in a context where people are expected to feel negative emotions, such as in this study (Maio and Esses, 2001; Leone and Presaghi, 2007; Appel et al., 2012). In the present research, we have not observed the matching effect for people with high levels of NFC and low levels of NFA. Literature about the structural matching effect has already shown a weaker matching effect for cognitive messages compared to the affective ones (Teeny et al., 2021). People with cognitive orientation, in fact, deeply process the arguments of the message to satisfy their cognitive needs and they are persuaded only by strong matched arguments (Petty and Wegener, 1998; Haddock et al., 2008). It is possible that, in the case of the present study, participants did not perceive Twitter users’ comments as arguments strong enough to satisfy this need.

For what concerns people with both high affective and high cognitive orientations, the slope difference test showed that they did not differ from those with high cognitive-low affective orientation and those with low cognitive-low affective orientation. Despite this, these participants’ attitudes were more negative when exposed to positive affective contents, while it was unaffected by cognitive ones. In people with high affective–high cognitive orientations and in a context in which the overall attitude toward a new lockdown is negative, it is possible to speculate that positive affective contents elicit dissonance resolved by amplifying the accessible (negative) response (Bell and Esses, 2002).

These novel findings contribute to research on attitude formation and persuasion, as well as health-related communication more broadly. From the attitudinal perspective, the present work represents a first attempt to test whether the persuasive effect of a message depends solely on the correspondence between people’s affective/cognitive orientation and the affective/cognitive content of the message (i.e., the classic matching effect) or whether it is also influenced by the valence of arguments not matching the individuals’ orientation. Results indicate that affective contents should be carefully considered when included in a health-related communication as they affect the attitudes of those who rely on them to create their evaluations. These findings confirmed the main findings in persuasive studies, which suggests the prevalence of affective–emotional appeals over cognitive ones in attitude change (e.g., Cacioppo and Petty, 1984; Rocklage et al., 2018). In line with these findings, persuasive matching research showed that affective matching (i.e., proposing an affective message to an affectively oriented individual) produces stronger persuasion effects than a cognitive matching (i.e., proposing cognitive messages to cognitively oriented individuals, see Teeny et al., 2021, for a review).

Moreover, it is worth noting that our findings confirmed how, in online communication, a persuasive effect is given by the comments and interactions to a post and not only by the post itself (see Dounoucos et al., 2019). For this reason, in online health campaigns is important to pay attention to comments in response to the message, not only to the message. The use of a single attitude object (lockdown) in a single specific context (Twitter) represents a limitation of the study. Nevertheless, the present research extends the recent evidence about the role of communication on social networks in the strategies of COVID-19 prevention (Raamkumar et al., 2020; Mori et al., 2021). It would be helpful for future research to generalize our results by considering objects unrelated to health issues and other social media in which comments can play an influential role (e.g., Facebook).

## Conclusion

The present work contributes to understanding the effects of the preference for affective or cognitive experiences in seeking out affective or cognitive information on the outcome of persuasive messages. Paying attention to content used to influence people’s attitudes has always been an important issue in social psychology, and this is particularly true when the object of the communication is related to health and the prevention of the spread of dangerous diseases. Therefore, we hope that those involved in spreading health-related persuasive messages and information will take into account the valence of the affective contents they use.

## Data availability statement

The raw data supporting the conclusions of this article will be made available by the authors, without undue reservation.

## Ethics statement

Ethical review and approval was not required for the study on human participants in accordance with the local legislation and institutional requirements. The patients/participants provided their written informed consent to participate in this study.

## Author contributions

All the authors contributed to the study’s conception and design, to design and test study materials, to collect and analyze the data, and to the manuscript writing and revision, and read and approved the submitted version.
