# Supplementary material for: The matching effect in persuasive communication about lockdown
Source: Front Psychol. 2022 Oct 12;13:987114. doi: 10.3389/fpsyg.2022.987114 (PMC9599401; doi:10.3389/fpsyg.2022.987114)
Supplement: Supplementary file 1 [file Data_Sheet_1.docx]

Supplementary Material

# Manipulation materials

Manipulation was realized through fabricated Twitter pages, one for each condition. Each of them was characterized by the same structure: a tweet affirming “*Un recente studio pubblicato su #Lancet (Womick & Roozenbeek, in press) suggerisce che la probabilità che nel prossimo anno si sviluppi una nuova variante del virus #COVID-19 resistente ai vaccini è molto alta. In questo caso un nuovo #lockdown totale sarà inevitabile.*” [A recent study published by #Lancet (Womick & Roozenbeek, in press) suggests that the likelihood of a new variant of the vaccine-resistant #COVID-19 virus developing in the next year is very high. In this case a new total #lockdown will be inevitable.], attributed to a Twitter profile named *COVID-19 Prevention Network*, accompanied by six comments.

## Original and translated materials

Find below the original (in Italian) positive cognitive comments with the related translation in square brackets:

- Considero vantaggioso il ricorso a un nuovo lockdown, perchè permetterebbe di non saturare i posti in ospedale [I think that a new lockdown would be beneficial, because it would allow to low the bed occupancy rate in hospitals];
- Trovo saggia l’idea di un nuovo lockdown quando necessario, è una misura che ha prodotto molti risultati positivi [I find it is wise to resort to a new lockdown when necessary, it is a measure that has produced many positive results];
- Penso che un nuovo lockdown sia davvero utile perchè i dati suggeriscono che è stato efficace nell’arginare il problema del contagio [I think that a new lockdown could be really useful because the data suggests it has been effective in limiting the contagion spread].

Find below the original (in Italian) positive affective comments with the related translation in square brackets:

- Tornerei tranquillamente in lockdown perchè potrei stare di più in compagnia dei miei cari [I would quietly accept to be in lockdown again because I can stay more in the company of my loved ones];
- Se dovesse essere confermata questa misura mi rilasserei perchè mi godrei i piccoli piaceri quotidiani[If this measure were to be confirmed, I would relax because I would enjoy the little daily pleasures];
- Sarei felice di tornare in lockdown perchè durante il primo mi sono sentito/a più rilassato del solito [I would be happy to be in lockdown again because during the first one I felt more relaxed than usual].

Find below the original (in Italian) negative cognitive comments with the related translation in square brackets:

- Penso che un nuovo lockdown sia inutile perchè crea più problemi di quanti ne risolve [I think a new lockdown is useless because it creates more problems than it solves];
- Considero dannoso il ricorso a un nuovo lockdown, perchè avrebbe un altissimo impatto economico negativo [I consider it is harmful resorting to a new lockdown, because it would have a strong negative economic impact];
- Penso che la misura del lockdown sia pericolosa, i dati hanno mostrato un aumento della disoccupazione [I think the lockdown measures are dangerous, the data showed an increase in unemployment]

Find below the original (in Italian) negative affective comments with the related translation in square brackets:

- Se dovesse essere confermata questa misura mi infurierei perché sarebbe una limitazione della mia libertà [If this measure were to be confirmed, I would be furious because it would limit my freedom];
- La prospettiva di un nuovo lockdown mi fa stare di malumore, dovrò rinunciare a molte cose [The prospect of a new lockdown makes me feel bad, I will have to give up many things];
- La prospettiva di un nuovo lockdown mi intristisce, mi sentirò molto solo/a [The prospect of a new lockdown saddens me, I will feel very lonely].
